# Protocol for a systematic review and individual patient data meta-analysis of benefit of so-called lung-protective ventilation settings in patients under general anesthesia for surgery

**DOI:** 10.1186/2046-4053-3-2

**Published:** 2014-01-02

**Authors:** Ary Serpa Neto, Sabrine NT Hemmes, Marcelo Gama de Abreu, Paolo Pelosi, Marcus J Schultz

**Affiliations:** 1 Department of Intensive Care, Academic Medical Center, University of Amsterdam, Meibergdreef 9, 1105 AZ Amsterdam, The Netherlands; 2 Laboratory of Experimental Intensive Care and Anesthesiology (LEICA), Academic Medical Center, University of Amsterdam, Meibergdreef 9, 1105 AZ Amsterdam, The Netherlands; 3 Medical Intensive Care Unit, ABC Medical School (FMABC), Lauro Gomes Avenue 2000, Santo André, Brazil; 4 Department of Critical Care Medicine, Hospital Israelita Albert Einstein, Albert Einstein Avenue, 700, São Paulo, Brazil; 5 Department of Anesthesiology, Academic Medical Center, University of Amsterdam, Meibergdreef 9, 1105 AZ Amsterdam, The Netherlands; 6 Department of Anesthesiology and Intensive Care Medicine, University Hospital Carl Gustav Carus, Fetscherstraße 74, 01307 Dresden, Germany; 7 Department of Surgical Sciences and Integrated Diagnostics, University of Genoa, Via Balbi 5, 16126 Genoa, Italy

**Keywords:** Surgery, Mechanical ventilation, Individual patient data, Protective ventilation

## Abstract

**Background:**

Almost all patients under general anesthesia for surgery need mechanical ventilation. The harmful effects of short-term intra-operative ventilation on pulmonary integrity are increasingly recognized. Recent investigations suggest protection against so-called ventilation-associated lung injury with the use of lower tidal volumes and/or the use of higher levels of positive end-expiratory pressure (PEEP). This review and meta-analysis will evaluate the effects of these protective measures on pulmonary and extra-pulmonary complications, and try to discriminate the effects of lower tidal volumes from those of higher levels of PEEP.

**Methods/design:**

The Medline database will be searched for observational studies and randomized controlled trials of intra-operative ventilation. Individual patient data will be collected from databases obtained via direct contact with corresponding authors of original articles. The primary endpoint is development of postoperative acute respiratory distress syndrome, the most important postoperative pulmonary complication. Secondary endpoints include hospital length of stay and hospital mortality, and reported intra-operative and postoperative pulmonary and extra-pulmonary complications. Emphasis is put on separating the effects of lower tidal volumes from those of higher levels of PEEP.

**Discussion:**

This will be the first meta-analysis of intra-operative ventilation using individual patient data from observational studies and randomized controlled trials. The large sample size could allow discrimination of the effect of the two most frequently used protective measures - that is, lower tidal volumes and higher levels of PEEP. The results of this review and meta-analysis can be used in designing future trials of ventilation.

## Background

Mechanical ventilation is frequently mandatory in patients under general anesthesia for surgery. The potential harmful effects of short-term intra-operative ventilation are increasingly recognized [1]. Ventilation can cause so-called ventilator-associated lung injury via overdistension of alveoli in aerated lung tissue, or repetitive opening and closing of atelectatic lung parts, or both. Alveolar overdistension could be prevented by use of lower tidal volumes [2], while higher levels of positive end-expiratory pressure (PEEP) could prevent against tidal recruitment [3].

Two conventional meta-analyses of observational studies and randomized controlled trials of intra-operative ventilation published at the beginning of 2013 suggest the use of so-called lung-protective ventilation with lower tidal volumes and/or higher levels of PEEP to prevent ventilator-associated lung injury and, as such, postoperative complications [4,5]. Two randomized controlled trials confirm this by showing that the combined use of lower tidal volumes and higher levels of PEEP protects against postoperative pulmonary complications [6,7]. However, these two trials were published after the publication of the meta-analyses and were not included in the final analyses.

It is not possible to conclude from the conventional meta-analyses or the two randomized controlled trials what was really responsible for the improved outcome: the use of lower tidal volumes or the use of higher levels of PEEP or the use of both. This is not an unimportant issue, since use of lower tidal volumes, but especially use of higher levels of PEEP, could be harmful as well – for example, by affecting intra-operative hemodynamics [8]. With the use of individual patient data, it may be possible to isolate the real effect of tidal volume from that of PEEP.

Some reports suggest that the ideal way to perform a meta-analysis is to use individual patient data [9]. First, use of individual patient data might overcome the power problem of individual trials and is therefore the gold standard for subgroup analyses. Also, the interaction effects between interventions, in this case the simultaneous use of lower tidal volumes and use of higher levels of PEEP, could be assessed and potential confounders can be adjusted for [9]. Therefore, the aim of this enterprise is to collect individual patient data from published observational studies and randomized controlled trials on protective intra-operative ventilation, and to evaluate the impact of the two different interventions on the development of postoperative complications. We hypothesize the protective effects of ventilation with lower tidal volume are more important than the protective effects of higher levels of PEEP.

## Methods/design

### Search strategy

Published and unpublished observational studies and randomized controlled trials were identified by two previous systematic searches of the literature by our group [4,5]. The sensitive search strategies followed Medical Subject Headings and Keywords ([*protective ventilation* OR *lower tidal volume* OR *low tidal volume*]). We will perform a new search strategy to assess studies dealing only with the effects of PEEP. Thus, we will include the following Medical Subject Heading and Keywords ([*protective ventilation* OR *lower tidal volume* OR *low tidal volume* OR *positive end*-*expiratory pressure* OR *positive end expiratory pressure* OR *PEEP*]). Two authors will perform a computerized blinded search of MEDLINE, Cumulative Index to Nursing and Allied Health Literature (CINAHL), Web of Science, and Cochrane Central Register of Controlled Trials (CENTRAL). The list of studies and trials will be updated to identify studies and trial published after the original searches, and thus updated to August 2013.

### Selection of studies

All observational studies and randomized controlled trials of protective ventilation that used outcomes relevant for this meta-analysis will be screened for inclusion. Key inclusion criteria are: 1) lower versus higher tidal volume in each arm, or higher versus lower PEEP in each arm, or both, 2) age > 18 years; 3) patients undergoing any surgical procedures under general anesthesia and mechanical ventilation; and 4) patients without acute respiratory distress syndrome (ARDS) at the onset of mechanical ventilation. Studies overlapping with other studies as indicated by the corresponding author will be excluded. There is no restriction in study design or language. Studies in patients in the ICU are also excluded.

### Methodological quality assessment

Two investigators will carry out data extraction and quality assessment from all the retrieved published studies based on the full text articles. Discrepancies will be resolved by consensus. In randomized controlled trials, we will assess allocation concealment, baseline similarity of groups (with regard to age, and severity of illness), early stopping of treatment, and loss to follow-up. Also, Jadad score will be used to assess the quality of the randomized controlled trials.

### Bias

We expect to obtain at least 90% or more of individuals analyzed in the studies identified in the search strategy. With this number of patients it will be possible to avoid the bias due to selective availability of study data. Sensitivity analysis combining the results of any unavailable studies (as extracted from publications or obtained in tabular form) and comparing these with the main individual patient data results will be used to interpret the data.

### Collection of individual patient data

Corresponding authors of the identified eligible published studies and trials will be contacted via email with a cover letter, or in a personal conversation, detailing the objectives of the collaborative meta-analysis, background information, and a datasheet for input of individual patient results for the project. The cover letter and the datasheet are shown in Additional file 1. The filled-out data templates will be sent back to the corresponding author and further communication will be mainly by email. Corresponding authors will also be contacted about unpublished data to enlarge the clinical data pool.

### Data management, security and validation

The same two investigators who perform the electronic database search will also collect and assemble individual patient data provided by the investigators. Data will be accepted in any kind of electronic format (SPSS, STATA, Word document, Excel document, and Access document) and only the coordinators of the collaboration will have direct access to the data. Both investigators will perform the data validation, checking the received data set for data entry mistakes and inconsistency. Differences will be discussed and settled in consensus.

### Mechanical ventilator parameters

The corresponding authors of studies and trials will be asked to fill the datasheet with mechanical ventilation parameters (plateau pressure, peak pressure, PEEP, respiratory rate, inspired fraction of oxygen (FiO_2_), and minute ventilation) and oxygenation parameters (partial pressure of oxygen (PaO_2_), partial pressure of carbon dioxide (PaCO_2_), pH, and the PaO_2_/FiO_2_ ratio) obtained hourly during the procedure.

### Analysis plan

The primary outcomes will be the occurrence of ARDS, according to the definition used by the authors, and the composite of ARDS development and in-hospital all-cause mortality. Secondary clinical outcomes include: 1) in-hospital mortality, defined as any death during hospital stay; 2) duration of mechanical ventilation, defined as the time since initiation of mechanical ventilation and successful discontinuation; 3) length of stay in the ICU, defined as the time from ICU admission to ICU discharge or death; 4) length of stay in hospital, defined as the time from hospital admission to hospital discharge or death; 5) occurrence of any intra-operative complication; and 6) occurrence of any postoperative pulmonary and extra-pulmonary complication, including pulmonary infections, according to the definition as used by the authors, need for red blood cell transfusions, defined as the amount of red blood cells in ml used during the follow-up, and need for fresh frozen plasma transfusion, defined as the amount of fresh frozen plasma in ml used during the follow-up. Secondary laboratorial outcomes will include: 1) levels of plasma IL-6, IL-8, IL-10, and TNF-α in pg/ml; and 2) levels of bronchoalveolar IL-6, IL-8, and TNF-α in pg/ml. Finally, safety outcomes will include: 1) lowest PaO_2_ in mmHg; 2) lowest and highest PaCO_2_ in mmHg; 3) worst PaO_2_/FiO_2_ ratio; 4) lowest and highest pH; and 5) incidence of acidosis, defined as pH <7.35.

### Completeness of data

Careful evaluation will be performed to ensure completeness of data and to check consistency. Since some authors may not have recorded ventilatory parameters hourly, we will divide the measurements into three periods: 1) beginning of the surgery, defined as the parameters measured in the first hour of the procedure; 2) middle of the surgery, defined as the parameters measured closest to the middle of the procedure (total time of procedure divided by two); and 3) end of the surgery, defined as the parameters measured in the last hour of the procedure. Since parameters at the end of the study can suffer influence of any lung injury developed during surgery and parameters at the beginning of surgery do not have sufficient time to induce changes in the lung, we will use the parameters in the middle of the surgery in the outcome analyses.

### Model selection

A multivariate model will be constructed for discrimination of the effects of lower tidal volume from those of higher levels of PEEP. The initial model will include age, gender, type of study, body mass index, type of surgery, (ASA) (American Society of Anesthesiology score), type of ventilation, highest PEEP used during surgery, highest plateau pressure achieved during surgery, highest compliance achieved during surgery, transfusion of red blood cells in the perioperative period, and risk factors for ARDS. Variables with *P* < 0.2 in the univariate analysis are included in the multivariate regression. The final model will be developed by dropping each variable in turn from the model and conducting a likelihood-ratio test to compare the full and the nested models. We will use a significance level of 0.05 as the cutoff to exclude a variable from the model. Finally, the variables of tidal volume (protective versus conventional in an intention-to-treat (ITT) analysis and ≤7 ml/kg predicted body weight (PBW) versus 7 to 10 ml/kg PBW versus >10 ml/kg PBW in a per protocol analysis) and PEEP (protective versus conventional in an ITT analysis and ≤5 cmH_2_O versus >5 cmH_2_O in a per protocol analysis) will be added to the model in order to test the resultant model against that without the variable. We will construct Kaplan-Meier curves and use log-rank test to determine the univariate significance of the study variables [9].

### Statistical analysis

Baseline characteristics of patients will be presented separately for each trial and overall. Continuous variables will be presented as mean ± SD or median and interquartile if not normally distributed. Binary and categorical variables will be presented as frequencies and percentages.

All analyses will be conducted in two ways: 1) an ITT analysis, where patients are analyzed according to the group of tidal volume they had in the original trial, taking into account only the randomized controlled trial; and 2) a per protocol analysis, where patients are analyzed according to the tidal volume they really received, taking into account the randomized controlled trial and observational studies. In ITT analyses the patients will be divided into two groups of tidal volume (protective versus conventional), and in per protocol analyses the patients will be divided into three groups of tidal volume (≤7 ml/kg PBW versus 7 to 10 ml/kg PBW versus >10 ml/kg PBW).

Time-to-event is defined as time from the day of surgery to the event. We will use a Cox proportional hazards regression model to examine simultaneously effects of multiple covariates on outcomes, censoring a patient’s data at the time of death, hospital discharge, or after 30 days [10]. In all models, the categorical variables will be tested for trend with the large tidal volume as reference and the proportional hazards assumption will be assessed. A test for interaction between pairs of variables in the final model will be performed. The effect of each variable in these models is assessed with the use of the Wald test and described by the hazard ratio with a 95% confidence interval. Binary outcomes will be analyzed by the chi-square test and by logistic regression including the same set of covariates as for the Cox proportional hazards regression model.

Restricted cubic spline analysis will be used to characterize the dose–response relationship between median tidal volume in ml/kg PBW and ARDS development, while adjusting for the same set of covariates as used in the final Cox model. A cubic or quadratic term will be used in the final model. Time-course variables (for example, repeated measures of ventilatory parameters, vital signs, oxygenation parameters and so forth) will also be analyzed by a linear mixed model. The linear mixed models procedure expands the generalized linear model so that the data are permitted to exhibit correlated and non-constant variability. The model includes two factors: 1) study group (fixed factor, defined as protective or conventional in the ITT analysis and tidal volume ≤7 ml/kg PBW versus 7 to 10 ml/kg PBW versus >10 ml/kg PBW in the per protocol analysis); each level of the study group factor can have a different linear effect on the value of the dependent variable; and 2) time as covariate; time is considered to be a random sample from a larger population of values, the effect is not limited to the chosen times.

Subgroup analyses will be used to assess the effects of tidal volume and PEEP in the following pre-specified subgroups: 1) type of surgery (cardiac, abdominal, thoracic, and orthopedic); 2) study design (randomized versus non-randomized controlled trial); 3) ASA score (<3 versus ≥3 and for each level); 4) presence of risk factor for ARDS; 5) mode of ventilation (volume versus pressure controlled); 6) age (<65 versus ≥65 years); 7) gender (male versus female); 8) PEEP during surgery (<5 versus ≥5 cmH_2_O); and 9) body mass index (<17, 18 to 25, 26 to 30, 31 to 35, and >35 kg/m^2^). For data not normally distributed, analyses will be performed after log_10_ transformation to permit the use of parametric statistics. If the data still differ significantly from normal even after log_10_ transformation, these data will be analyzed by non-parametric tests.

All analyses will be conducted with Review Manager v.5.1.1 (The Nordic Cochrane Centre, The Cochrane Collaboration, Copenhagen, Denmark), SPSS v.20 (IBM Corporation, New York, USA) and R v.2.12.0 (R Foundation for Statistical Computing, Vienna, Austria). For all analyses two-sided *P* values <0.05 will be considered significant.

### Publication policy

This protocol is not registered in the PROSPERO. The results of this meta-analysis will be sent for publication in a peer-reviewed journal and all collaborators will be included as co-authors of the paper.

## Discussion

The individual patient data meta-analysis as proposed here is necessary to assess the effect of protective ventilation settings in patients under anesthesia for surgery. This is the first meta-analysis that will assess and combine data from all studies and trials of protective ventilation during surgery. Factors that influence the incidence of postoperative complications can be identified in this meta-analysis. Finally, this individual patient meta-analysis may help anesthesiologists to improve ventilation settings in patients in the operating room.

Protective mechanical ventilation using lower tidal volumes has been proven to reduce ventilator-associated lung injury. The use of lower tidal volumes has been found beneficial in patients who needed long-term mechanical ventilation for ARDS [11,12], showing a reduction of mortality as high as 9%. One randomized controlled trial [13] and a recent meta-analysis [4] suggest lower tidal volume to be beneficial also in patients without ARDS. Several smaller studies and trials [6,7] and two recent meta-analyses [4,5] suggest that protective ventilation is also beneficial in short-term ventilation for patients during general surgery. However, in all trials, different levels of PEEP were used, making comparison and interpretation of the individual effect of PEEP and tidal volume on postoperative outcome difficult.

In critically ill patients under mechanical ventilation in the ICU, the use of low tidal volume can increase the use of sedatives and muscle relaxants increasing the incidence of ICU delirium and ICU-acquired weakness. Both conditions have the potential to lengthen duration of mechanical ventilation and stay in ICU. Furthermore, it is argued that use of lower tidal volumes is not always possible with spontaneous modes of ventilation, which are most frequently used in ICU patients without ARDS [14]. Also, higher levels of PEEP are associated with hemodynamic impairment and with the risk of barotrauma [8]; thus, it is important to understand the benefits of these strategies in the face of their risks.

In conclusion, with this individual patient data meta-analysis we will be able to assess the independent effects of different so-called lung-protective ventilation settings in (subgroups of) patients under general anesthesia for surgery.

## Abbreviations

ARDS: Acute respiratory distress syndrome; ASA: American Society of Anesthesiology score; FiO2: Inspired fraction of oxygen; IL: Interleukin; ITT: Intention-to-treat; PaCO2: Partial pressure of carbon dioxide; PaO2: Partial pressure of oxygen; PBW: Predicted body weight; PEEP: Positive end-expiratory pressure; TNF: Tumor necrosis factor.

## Competing interests

The authors declare that they have no competing interests.

## Authors’ contributions

ASN, SNTH, MGdA, PP and MJS conceived the idea, and drafted the initial protocol. All authors reviewed, commented on, read and approved the final manuscript.

## Supplementary Material

Additional file 1 The cover letter sent to corresponding authors and the datasheet for individual data collection. Click here for file

## References

[B1] Schultz MJ Haitsma JJ Slutsky AS Gajic O What tidal volumes should be used in patients without acute lung injury? Anesthesiology 2007 106 1226 1231 10.1097/01.anes.0000267607.25011.e8 17525599

[B2] Dreyfuss D Saumon G Ventilator-induced lung injury - lessons from experimental studies Am J Respir Crit Care Med 1998 157 294 323 10.1164/ajrccm.157.1.9604014 9445314

[B3] Lu Q How to assess positive end-expiratory pressure-induced alveolar recruitment? Minerva Anestesiol 2013 79 83 91 23135694

[B4] Serpa Neto A Cardoso SO Manetta JA Pereira VG Espósito DC Pasqualucci Mde O Damasceno MC Schultz MJ Association between use of lung-protective ventilation with lower tidal volumes and clinical outcomes among patients without acute respiratory distress syndrome: a meta-analysis JAMA 2012 308 1651 1659 10.1001/jama.2012.13730 23093163

[B5] Hemmes SN Serpa Neto A Schultz MJ Intraoperative ventilatory strategies to prevent postoperative pulmonary complications: a meta-analysis Curr Opin Anaesthesiol 2013 26 126 133 10.1097/ACO.0b013e32835e1242 23385321

[B6] Futier E Constantin JM Paugam-Burtz C Pascal J Eurin M Neuschwander A Marret E Beaussier M Gutton C Lefrant JY Allaouchiche B Verzilli D Leone M De Jong A Bazin JE Pereira B Jaber S IMPROVE Study Group A trial of intraoperative low-tidal-volume ventilation in abdominal surgery N Engl J Med 2013 369 428 437 10.1056/NEJMoa1301082 23902482

[B7] Severgnini P Selmo G Lanza C Chiesa A Frigerio A Bacuzzi A Dionigi G Novario R Gregoretti C de Abreu MG Schultz MJ Jaber S Futier E Chiaranda M Pelosi P Protective mechanical ventilation during general anesthesia for open abdominal surgery improves postoperative pulmonary function Anesthesiology 2013 118 1307 1321 10.1097/ALN.0b013e31829102de 23542800

[B8] Hemmes SN Severgnini P Jaber S Canet J Wrigge H Hiesmayr M Tschernko EM Hollmann MW Binnekade JM Hedenstierna G Putensen C de Abreu MG Pelosi P Schultz MJ Rationale and study design of PROVHILO - a worldwide multicenter randomized controlled trial on protective ventilation during general anesthesia for open abdominal surgery Trials 2011 12 111 10.1186/1745-6215-12-111 21548927 PMC3104489

[B9] Clarke MJ Stewart LA Obtaining data from randomised controlled trials: how much do we need for reliable and informative meta-analyses? BMJ 1994 309 1007 1010 10.1136/bmj.309.6960.1007 7950694 PMC2541306

[B10] Cox DR Regression models and life-Tables J R Stat Soc [B] 1972 34 187 220

[B11] Acute Respiratory Distress Syndrome Network Ventilation with lower tidal volumes as compared with traditional tidal volumes for acute lung injury and the acute respiratory distress syndrome N Engl J Med 2000 342 1301 1308 10793162 10.1056/NEJM200005043421801

[B12] Putensen C Theuerkauf N Zinserling J Wrigge H Pelosi P Meta-analysis: ventilation strategies and outcomes of the acute respiratory distress syndrome and acute lung injury Ann Intern Med 2009 151 566 576 10.7326/0003-4819-151-8-200910200-00011 19841457

[B13] Determann RM Royakkers A Wolthuis EK Vlaar AP Choi G Paulus F Hofstra JJ de Graaff MJ Korevaar JC Schultz MJ Ventilation with lower tidal volumes as compared with conventional tidal volumes for patients without acute lung injury: a preventive randomized controlled trial Crit Care 2010 14 R1 10.1186/cc8230 20055989 PMC2875503

[B14] Ferguson ND Low tidal volumes for all? JAMA 2012 308 1689 1690 10.1001/jama.2012.14509 23093167

