# Supplementary material for: Protocol for a systematic review and individual patient data meta-analysis of benefit of so-called lung-protective ventilation settings in patients under general anesthesia for surgery
Source: Syst Rev. 2014 Jan 2;3:2. doi: 10.1186/2046-4053-3-2 (PMC3880983; doi:10.1186/2046-4053-3-2)
Supplement: Additional file 1 — The cover letter sent to corresponding authors and the datasheet for individual data collection. [file 2046-4053-3-2-S1.docx]

**A PROTOCOL FOR A SYSTEMATIC REVIEW AND INDIVIDUAL PATIENT DATA META-ANALYSIS OF PROTECTIVE VENTILATION IN PATIENTS UNDER GENERAL ANESTHESIA FOR SURGERY**

ONLINE ONLY DATA

Ary Serpa Neto MD MSc, Sabrine NT Hemmes MD, Marcelo Gama de Abreu MD PhD, Paolo Pelosi MD PhD, Marcus J Schultz MD PhD

**APPENDIX 1**

Cover letter sent to corresponding authors

Dear Dr.

Hope this e-mail finds you well.

My name is Ary Serpa Neto and I'm a researcher from São Paulo / Brazil. Recently our group reported on a systematic review and meta-analysis testing the hypothesis whether use of lower tidal volumes benefits patients without lung injury at the onset of mechanical ventilation (JAMA 2012;308(16)1651-9). Your paper entitled "XXXX. (XXXX. 2012 Jun;24(4):263-9)" was of significant importance in this topic. Now, we are planning to analyze the data in another way, and the plan is to conduct a meta-analysis of individual patient data.

On behalf of my group and of Prof. Dr. Marcus J. Schultz we kindly invite you to participate in this project; your assignments will be to provide the individual data of your study for the calculations, review and drafting of the paper and, in the final, joining us as a co-author of the paper. We believe that this paper will result in a good publication (as we already done with a "conventional" meta-analysis in JAMA).

Your help is of great importance, and the results of the meta-analysis may be useful for future studies

Kindest regards,

Ary Serpa Neto, MD, MSc

Marcus J Schultz, MD, PhD

**APPENDIX 2**

Datasheet for individual data collection

**1. General information**

1.1. Total number of patients in each arm

1.2. Corresponding author

1.3. Type of study, randomized controlled trial or observational

1.4. Group, protective or conventional

**2. Demographic data**

2.1. Age, years

2.2. Gender, male/female

2.3. Body mass index, kg/m^2^

2.4. ASA score

2.5. Height, cm

2.6. Weight, kg

2.7. Predicted body weight, kg

2.8. Type of surgery

2.9. Risk factors for ARDS

2.9.1. Sepsis

2.9.2. Transfusion

2.9.3. Shock

2.9.4. Pneumonia

**3. Mechanical ventilation parameters (measured hourly or at the beginning and/or middle and/or finish of the surgery)**

3.1. Type of mechanical ventilation, volume controlled or pressure controlled

3.2. Tidal volume, mL and mL/kg PBW

3.3. Peak pressure, cmH_2_O

3.4. Plateau pressure, cmH_2_O

3.5. Positive end expiratory pressure, cmH_2_O

3.6. Respiratory rate, bpm

3.7. Minute-ventilation, L/min

3.8. Inspired fraction of oxygen, %

**4. Oxygenation parameters (measured hourly or at the beginning and/or middle and/or finish of the surgery)**

4.1. Partial pressure of oxygen, mmHg

4.2. Partial pressure of carbon dioxide, mmHg

4.3. pH

**5. Clinical outcomes**

5.1. ARDS development, %

5.2. In-ICU mortality, %

5.3. In-hospital mortality, %

5.4. Barotrauma, %

5.5. Time of mechanical ventilation, minutes

5.6. Pulmonary infection, %

5.7. ICU length of stay, days

5.8. Hospital length of stay, days

5.9. Red blood cell transfusion, mL

5.10. Fresh frozen plasma transfusion, mL

**6. Laboratorial outcomes (measured the beginning and/or middle and/or finish of the surgery)**

6.1. Plasma interleukin-6, pg/ml

6.2. Plasma interleukin-8, pg/ml

6.3. Plasma interleukin-10, pg/ml

6.4. Plasma TNF-α, pg/ml

6.5. Bronchoalveolar interleukin-6, pg/ml

6.6. Bronchoalveolar interleukin-8, pg/ml

6.7. Bronchoalveolar TNF-α, pg/ml
